# Heritability Estimation Approaches Utilizing Genome‐Wide Data

**DOI:** 10.1002/cpz1.734

**Published:** 2023-04-17

**Authors:** Amit K. Srivastava, Scott M. Williams, Ge Zhang

**Affiliations:** ^1^ Division of Human Genetics Cincinnati Children's Hospital Medical Center Cincinnati Ohio; ^2^ The Center for Prevention of Preterm Birth, Perinatal Institute Cincinnati Children's Hospital Medical Center Cincinnati Ohio; ^3^ March of Dimes Prematurity Research Center Ohio Collaborative Cincinnati Ohio; ^4^ Department of Pediatrics University of Cincinnati College of Medicine Cincinnati Ohio; ^5^ Department of Population and Quantitative Health Sciences, School of Medicine Case Western Reserve University Cleveland Ohio; ^6^ Department of Genetics and Genome Sciences, School of Medicine Case Western Reserve University Cleveland Ohio; ^7^ Institute of Computational Biology Case Western Reserve University Cleveland Ohio

**Keywords:** individual‐level data, SNP‐heritability, summary results

## Abstract

Prior to the development of genome‐wide arrays and whole genome sequencing technologies, heritability estimation mainly relied on the study of related individuals. Over the past decade, various approaches have been developed to estimate SNP‐based narrow‐sense heritability ($h_{\mathrm{SNP}}^{2}$) in unrelated individuals. These latter approaches use either individual‐level genetic variations or summary results from genome‐wide association studies (GWAS). Recently, several studies compared these approaches using extensive simulations and empirical datasets. However, sparse information on hands‐on training necessitates revisiting these approaches from the perspective of a stepwise guide for practical applications. Here, we provide an overview of the commonly used SNP‐heritability estimation approaches utilizing genome‐wide array, imputed or whole genome data from unrelated individuals, or summary results. We not only discuss these approaches based on their statistical concepts, utility, advantages, and limitations, but also provide step‐by‐step protocols to apply these approaches. For illustration purposes, we estimate $h_{\mathrm{SNP}}^{2}$ of height and BMI utilizing individual‐level data from The Northern Finland Birth Cohort (NFBC) and summary results from the Genetic Investigation of ANthropometric Traits (GIANT;) consortium. We present this review as a template for the researchers who estimate and use heritability in their studies and as a reference for geneticists who develop or extend heritability estimation approaches. © 2023 The Authors. Current Protocols published by Wiley Periodicals LLC.

**Basic Protocol 1**: GREML (GCTA)

**Alternate Protocol 1**: Stratified GREML

**Basic Protocol 2**: LDAK

**Alternate Protocol 2**: Stratified LDAK

**Basic Protocol 3**: Threshold GREML

**Basic Protocol 4**: LD score (LDSC) regression

**Basic Protocol 5**: SumHer

## INTRODUCTION

A long‐standing question in quantitative and behavioral genetics is whether the variation in a particular trait is due to genetic or environmental factors (Visscher et al., 2008). A key step in finding an answer to this question is partitioning the observed phenotypic variance into variance components attributable to unobserved genetic and environmental factors. R. A. Fisher (Fisher, 1918) first modeled and partitioned the phenotypic variance into genetic and environmental components without any knowledge of specific genes affecting the trait (Visscher & Goddard, 2019). Although he did not use the term ‘heritability’, his research laid the foundation for various future approaches for the estimation of heritability (Falconer, 1960; Walsh, 1998).

Heritability is defined as the proportion of phenotypic variance that is attributable to genetic factors in a given population at a specific time(Falconer, 1960; Walsh, 1998). Heritability can be defined in two ways. The broad‐sense heritability (H^2^) estimates the proportion of phenotypic variance attributable to all genetic factors, including additive genetic effects (A), dominant genetic effects (D), and epistatic effects (G × G) (Mayhew & Meyre, 2017; Visscher et al., 2008; Zhu & Zhou, 2020). In contrast, narrow‐sense heritability (h^2^) estimates the proportion of phenotypic variance attributable to additive genetic effects (A) or breeding values (Mayhew & Meyre, 2017; Visscher et al., 2008; Zhu & Zhou, 2020). Since h^2^ is more relevant to the evaluation of genetic influence on phenotypic resemblance of relatives and predicting evolutionary responses to selection, it is a commonly used parameter for heritability estimation and applications (Visscher et al., 2008).

Heritability plays an important role in several areas of biology such as agriculture, medicine, and evolution (Visscher et al., 2008). It facilitates selective breeding programs to improve the quality of plant and domestic animals (Alvarez, 2017; Bernardo, 2020; Berry et al., 2003, 2014; Cassell, 2009; Manjula et al., 2018; Miglior et al., 2017; Palmquist & Jenkins, 2017; Utrera & Van Vleck, 2004; Velasco & Fernández‐martínez, 2002). Heritability also provides insights into the genetic architecture of complex traits and diseases (Eichler et al., 2010; Friedman et al., 2021; Lunde et al., 2007; Manolio et al., 2009; Silventoinen et al., 2003; Tenesa & Haley, 2013; Vinkhuyzen et al., 2013; Visscher et al., 2007; Wray et al., 2007). For over a century, heritability has played a key role in measuring the genetic influence on various traits and diseases (Dempster & Lerner, 1950; Falconer, 1960, 1965). A large heritability implies that genetic factors have a strong influence on a trait or disease. Being an important parameter of genetic influence on phenotype, heritability has been frequently used as the basis for genetic linkage and genetic association studies (Boomsma et al., 2002; Friedman et al., 2021; Institute of Medicine, 2006). These studies led to the discovery of genes associated with various anthropometric and behavioral traits and diseases such as birth defects, psychiatric disorders, etc. (Institute of Medicine, 2006; Visscher et al., 2008). Therefore, an accurate estimation of heritability can help prioritize the use of resources for further genetic studies (Zhu & Zhou, 2020). Heritability acts as a key for understanding the evolution of quantitative traits and diseases (Bateson, 1922; Fisher, 1930; Grant & Grant, 1995; Hadfield, 2008; Kelly, 2011; Kingsolver et al., 2001; Lande & Arnold, 1983; Mousseau & Roff, 1987; Wood et al., 2016). Particularly, heritability determines how a population will respond to selection. Therefore it can be utilized to compare the evolution of a particular trait or disease across different populations at the same time and within a population at different timepoints (Mayhew & Meyre, 2017).

To date, aspects of heritability such as its conceptualization and applications (Powell et al., 2010; Visscher et al., 2008, 2010; Wray et al., 2013; Yang et al., 2017), assessments of missing heritability (Brookfield, 2013; Eichler et al., 2010; Genin, 2020; Golan et al., 2014; Manolio et al., 2009; Maroilley & Tarailo‐Graovac, 2019; Tenesa & Haley, 2013; Yang et al., 2011; Zaitlen & Kraft, 2012), methods and approaches (Boomsma et al., 2002; Browning & Browning, 2012; Evans, Tahmasbi, Jones, et al., 2018; Friedman et al., 2021; Hall & Bush, 2016; Pasaniuc & Price, 2017; Powell et al., 2010; Speed & Balding, 2015; Speed et al., 2020; VanRaden, 2008; Weir et al., 2006; Yang, Manolio et al., 2013; Zaitlen & Kraft, 2012), statistical models for various data types (Zhang et al., 2021; Zhu & Zhou, 2020) have been addressed in many reviews. In particular, heritability estimation methods and approaches have been key to many of these reviews irrespective of their central theme. These approaches depend on either the expected genetic similarity in pedigrees, e.g., family‐based approaches (Allison et al., 1996; Boomsma et al., 2002; Eaves et al., 1978; Falconer, 1960; Lunde et al., 2007; Nance et al., 1983; Stunkard et al., 1990; Walsh, 1998; Wright, 1921), or the realized genetic similarity among individuals in a population of mixed relationships—e.g., population‐based approaches (Browning & Browning, 2012; Lee & van der Werf, 2006; Lee et al., 2010; Ritland, 1996, 2000; Thomas, 2005; VanRaden, 2008; Yang et al., 2010, 2014, 2017; Zhang et al., 2010). Population‐based approaches generally utilize single nucleotide polymorphisms (SNPs) to estimate realized genetic similarity, and are usually called SNP‐heritability estimation approaches (Speed et al., 2012; Tang et al., 2022; Tenesa & Haley, 2013; Yang, Lee, et al., 2011; Yang et al., 2014; Yang, Manolio, et al., 2011; Zhu & Zhou, 2020).

**Figure 1:**
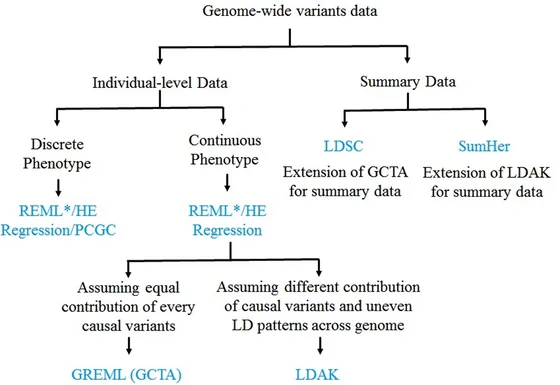
A summary of SNP‐heritability estimation approaches utilizing individual‐level genome‐wide SNPs or summary results from previous GWAS. Such data could be generated through array, imputation, and whole genome sequencing. REML, Restricted Maximum Likelihood Method; PCGC, Phenotype Correlation‐Genotype Correlation; HE, Hasemann‐Elston Regression; GREML, Genomic Restricted Maximum Likelihood; LDAK, Linkage Disequilibrium adjusted Kinship; LDSC, LD Score Regression.

New approaches to estimate heritability (VanRaden, 2008; Yang et al., 2010) were developed in parallel with advanced genotyping and sequencing technologies (1000 Genomes Project Consortium et al., 2010, 2012, 2015; International HapMap, 2005) that facilitated the estimation of realized genetic similarity; these approaches became popular for estimating SNP‐heritability in natural populations, as they did not require large pedigree recruitment. In the last decade, several approaches have been developed for estimation of SNP‐heritability of complex human traits and diseases. These approaches utilize either individual‐level genetic variations such as genome‐wide complex trait analysis (GCTA; Yang et al., 2010; Yang, Lee, et al., 2011), linkage‐disequilibrium‐adjusted kinships (LDAK; Speed et al., 2012, 2017), threshold genome‐based restricted maximum likelihood (Threshold GREML; Zaitlen et al., 2013), or summary results from GWAS such as LD Score (LDSC) regression (Bulik‐Sullivan et al., 2015; Zaitlen et al., 2013) and SumHer (Speed & Balding, 2019) (Fig. 1). Recently, several studies compared SNP‐heritability estimation approaches using simulated and empirical datasets (Evans, Tahmasbi, Vrieze, et al., 2018; Hou et al., 2019; Speed et al., 2017, 2020; Tang et al., 2022; Uricchio, 2020; Yang et al., 2017; Zhu & Zhou, 2020). However, few resources provide hands‐on training for these various approaches, thereby warranting an overview along with step‐by‐step protocols for practical applications.

The current review provides an updated summary of SNP‐heritability estimation approaches utilizing either individual‐level genome‐wide data or summary results from previous GWAS. These approaches utilize a variety of methods such as Maximum Likelihood (ML; Thompson, 1971; Visscher et al., 2006), Restricted Maximum Likelihood (REML; Lee & van der Werf, 2006; Yang et al., 2010), Haseman‐Elston (HE) Regression (Haseman & Elston, 1972; Sham & Purcell, 2001), and Phenotype Correlation–Genotype Correlation (PCGC; Golan et al., 2014). REML is the most widely used method for individual‐level genetic data, and is employed in linear mixed model (LMM) to simultaneously estimate the contribution of fixed and random effects. Therefore, we focus here on the REML methods while discussing the approaches developed for individual‐level genetic data. Likewise, we discuss LDSC (Bulik‐Sullivan et al., 2015), and SumHer (Speed & Balding, 2019) that utilize the regression method and REML, respectively, to estimate SNP‐heritability from GWAS summary results. We discuss each heritability estimation approach in the context of statistical basis, utility, advantages, and limitations. We also provide stepwise protocols to apply commonly used approaches utilizing individual‐level genetic data such as GCTA (Yang et al., 2010; Yang, Lee, et al., 2011), LDAK (Speed et al., 2012, 2017), and Threshold GREML (Zaitlen et al., 2013), as well as GWAS summary results such as LDSC (Bulik‐Sullivan et al., 2015; Bulik‐Sullivan et al., 2015), and SumHer (Speed & Balding, 2019). We present this review as a template to the researchers who need to estimate and use heritability in their research and as a reference to the geneticists who want to develop or extend heritability estimation approaches.

## AN OVERVIEW OF SNP‐HERITABILITY ESTIMATION APPROACHES

Genome‐wide SNP arrays and whole genome sequencing technologies have revolutionized many aspects of human genetics such as determination of genetic susceptibility and underlying mechanisms that increase risk of diseases, estimation of heritability, and understanding the evolution of complex traits (Eichler et al., 2010; Visscher et al., 2008; Zaitlen & Kraft, 2012). Genome‐wide association studies (GWAS) have discovered a multitude of genetic variants associated with various complex human traits and diseases (Buniello et al., 2019). However, variants derived from GWAS explain only a small proportion of phenotypic variance as compared to family studies, leading to a major question of missing (hidden) heritability (Manolio et al., 2009). There are several possible reasons for missing heritability, such as weak linkage disequilibrium (LD) between genotyped variants and ungenotyped causal variants, common variants with small effects that do not reach the canonical significance threshold (5 × 10^–8^) in GWAS, rare variants with large effects not captured by genotyping arrays, contribution of non‐additive effects, gene–environmental interactions, and overestimation of narrow‐sense heritability in pedigree‐based studies due to shared environmental confounding (Eichler et al., 2010; Gibson, 2012; Manolio et al., 2009; Yang et al., 2017; Zhang, 2015). In last decade, several approaches have been developed that utilize genome‐wide variations instead of only statistically significant variations to solve the problem of missing heritability of complex traits (Bulik‐Sullivan et al., 2015; Speed & Balding, 2019; Speed et al., 2012, 2017; Yang, Lee, et al., 2011; Zaitlen et al., 2013). These approaches successfully explained a large proportion of phenotypic variance attributable to genome‐wide SNPs for a variety of complex human traits and diseases. Unlike widely studied continuous traits such as anthropometric traits, behavioral traits, and pre‐ and perinatal traits, dichotomous traits such as diseases are represented on a discrete scale (e.g., 0‐1). Therefore, observed heritability on a risk scale is usually parameterized on an unobserved continuous liability scale so that the heritability is independent of disease prevalence (Falconer, 1965; Lee et al., 2011; Tenesa & Haley, 2013; Yang et al., 2017). Here, we explain major advances in the approaches based on genome‐wide SNP data at individual or summary level with their advantages and limitations (Table 1).

**Table 1:** Summary of Widely Used Approaches for the Estimation of SNP‐Heritability^*a*^

Approach	Statistical assumptions	Description	Advantages	Limitations
**GREML‐SC**	(i) Normal distribution of SNP effects $\left[u_{i}∼{\;N(0,h}_{g}^{2}/m)\right]$, independent of LD and inversely proportional to MAF; (ii) Polygenic model; (iii) Uncorrelated genetic and environmental components.	(i) Each SNP contributes equally to phenotypic variance i.e., $\sigma_{i}^{2}=h_{g}^{2}/m$; ii) ${\hat{h}}_{\mathrm{SNP}}^{2}$ is dependent on the tagging of causal variants by the SNPs used to create the GRM.	First ever approach to estimate SNP‐heritability using genome‐wide data in unrelated individuals.	(i) Highly dependent on LD among assayed and causal variants and biased to the extent to which the average LD among causal variants differ from the average LD among SNPs used to create GRM. (ii) No flexibility of modeling uneven LD and MAF influence as compared to other contemporary approaches.
**GREML‐MS**	Each GRM should follow same assumptions as GREML‐SC. SNP effects follow the distribution $\left[u_{i}∼\;N(0,\;\sum_{s\;\varepsilon \;m}h_{s}^{2}/s)\right]$.	(i) Multi‐component GREML—multiple GRMs based on MAF bins are fitted simultaneously in the linear mixed model (LMM); (ii) GRMs based on variety of bins such as chromosomes, genomic regions, functional annotation can be used.	Creating GRMs based on MAF bins can address the influence of MAF on SNP effects and variance. Since LD depends on MAF, GREML‐MS can resolve uneven tagging of causal variants up to some extent.	(i) Biased when LD structure of causal variants differ from that of the SNPs used to create the GRM; (ii) Relatively large standard errors.
**GREML‐LDMS‐R**	Same as GREML‐MS.	Multi‐component GREML that bins SNPs by their MAF and regional LD scores.	Same as GREML‐MS with additional advantage due to LD bins.	(i) Similar to GREML‐MS—if regional LD scores of causal variants differ from surrounding SNPs used to create GRM; (ii) Relatively large standard errors.
**GREML‐LDMS‐I**	Same as GREML‐MS.	Multi‐component GREML that bins SNPs by their MAF and individual LD scores.	To date, best version of GREML (least biased approach) which can address the uneven tagging of causal variants and the influence of MAF on SNP effects.	(i) Relatively large standard errors; (ii) Usually runs 20 genetic components, therefore, difficult to constrain REML (0 < ĥ^2^ < 1), particularly when ĥ^2^ and/or sample size is small.
**LDAK**	Same as GREML‐SC, except that (i) contribution of causal variants are different depending on their LD with surrounding SNPs. LDAK allows modeling of uneven LD patterns across genome via weighing thinned SNPs differently and the influence of MAF on SNP effects. $u_{i}∼N\left(0,\sigma_{i}^{2}\right);$ $\sigma_{i}^{2}\propto w_{i}{\left[p_{i}\left(1-p_{i}\right)\right]}^{1+\alpha }$	Developed to address the problem of uneven tagging of causal variants by the SNPs used to create GRM. Recommends using α = −0.25.	Can correct uneven tagging of causal variants and allows modeling the influence of MAF on SNP effects.	(i) As biased as GREML‐SC if assumptions aren't met; (ii) generally, larger standard errors as compared to GREML.
**LDAK‐MS**	Each GRM must hold same assumptions as LDAK.	Multi‐component version of LDAK that bins SNPs by MAF.	Developed to give flexibility of fitting various models based on MAF bins.	(i) Less biased than LDAK, but more biased than GREML‐LDMS; (ii) Relatively large standard errors.
**Threshold GREML**	Estimates associated with the GRM without threshold are like GREML‐SC. Variance attributable to the GRM with threshold represents ($h_{\mathrm{ped}}^{2}$ ‐ $h_{\mathrm{SNP}}^{2}$); where $h_{\mathrm{ped}}^{2}$ is the sum of variance attributable to both GRMs whereas $h_{\mathrm{SNP}}^{2}$ is the variance attributable to the GRM without threshold.	Multi‐component GREML with two GRMs: first GRM is created from all SNPs and second GRM is created by setting off‐diagonals below a set threshold to 0.	Generally useful in samples with extended genealogy.	It can be upwardly biased by shared environmental influences.
**LD score (LDSC) regression**	Polygenic model with normally distributed SNP effects. Statistical assumptions are same as GREML $[E\left[u_{i}\right]=0;$ $\mathrm{var}\left(u_{i}\right)={\;h}_{g}^{2}/m]$.	Slope from regression of χ^2^ (from GWAS) on SNPs’ LD scores (from reference data) is used to estimate h^2^ attributable to the causal variants in LD with common SNPs present in GWAS summary result.	(i) As compared to GREML, it requires only summary results instead of individual‐level data; (ii)Besides estimation of h^2^, LDSC can estimate genetic correlation with other traits; (iii) LDSC was further extended to estimate h^2^ attributable to various functional annotations, cell and tissue types (Finucane et al., 2015); (iv) Generally robust to confounding due to stratification and shared environmental effects; (v) computationally efficient.	(i) Estimated h^2^ is attributed to common causal variants only; (ii) Underestimates h^2^ if the trait is not highly polygenic; (iii) Biased estimates of h^2^ if reference population differs from the population used in GWAS.
**SumHer**	Basic idea is like LDSC with three differences: (i) SumHer models inflation as multiplicative whereas LDSC models as additive; (ii) Unlike LDSC, SumHer allows modeling uneven LD patterns across genome as well as influence of MAF on SNP effects; (iii) SumHer uses REML instead of regression to estimate SNP‐heritability.	An extension of the LDAK model that can estimate h^2^ from summary results of previous GWAS. It can also partition h^2^_g_ attributable to different annotations.	Multiplicative modeling of inflation can be useful to avoid overcorrecting confounding in large GWAS; (ii) SumHer has striking difference from LDSC in estimating h^2^ attributable to annotated regions.	Same as LDSC, except that SumHer apparently overestimates h^2^.

^*a*^ Each approach is summarized on the basis of statistical assumptions and concept along with their advantages and limitations. u_i_, p_i_, w_i_, and $\sigma_{i}^{2}$ represent effect, MAF, weight and variance of SNP i, respectively; m represents number of SNPs used to create GRM or number of SNPs in summary statistics; s represents a subset of m present in a MAF bin, LD bin, genomic region or functional annotation; $h_{g}^{2}$ and $h_{s}^{2}$ represent SNP‐heritability attributable to all SNPs used in the analysis and s subset of SNPs, respectively; α is a scaling factor, representing the influence of MAF on the variance of SNP effect.

### Approaches Utilizing Individual‐Level Genetic Data

The fundamental idea behind the approaches developed for individual‐level genome‐wide data is to estimate the realized genetic relationship among individuals by using genome‐wide variants and using this relationship matrix to estimate the genetic variance. Yang et al. (2010) first utilized such approach to address the problem of missing heritability of human height. The study used 294,831 SNPs genotyped on 3925 unrelated individuals to calculate realized genetic relatedness and fitted this relatedness matrix in LMM to estimate SNP‐based narrow‐sense heritability (${\hat{h}}_{\mathrm{SNP}}^{2}$) of human height. The results explained 45% of phenotypic variance in human height. Here, we discuss the most widely used SNP‐heritability estimation approaches utilizing individual‐level genetic data.

#### Genome‐wide Complex Trait Analysis (GCTA)

One of the most popular software packages for estimating SNP‐heritability using genome‐wide data from unrelated individuals is Genome‐wide Complex Trait Analysis (GCTA), which uses a genome‐based restricted maximum likelihood (GREML; Yang et al., 2010; Yang, Lee, et al., 2011) method. GCTA depends upon LD between genotyped variants and ungenotyped causal variants to estimate additive genetic variance in unrelated individuals.

The basic concept behind the method is to fit the effects of all the SNPs as random effects via an LMM. In this design, phenotype Y can be represented in simple equation form: Y=Zu+e, where Z is standardized genotype matrix (scaled genotypic values from unrelated individuals), u is the vector of random genetic effects with $u_{i}∼N\left(0,\sigma_{i}^{2}\right)$) for SNP i, and e is the vector of residual effects [e ∼ N(0, I$\sigma_{e}^{2}$)]. This model can be rewritten as Y=g+e, g = Zu is the additive genetic values of phenotype Y [g ∼ N(0, ${A\sigma }_{g}^{2}$)], A is the genetic relatedness matrix (GRM) among unrelated individuals ($A=\frac{ZZ^{T}}{m}$, m is the number of variants), and $\sigma_{g}^{2}$ is additive genetic variance of all variants ($\sigma_{g}^{2}={m\sigma }_{i}^{2}$). Similarly, phenotypic variance of Y can be expressed in terms of variance attributable to random (additive) effects ($\sigma_{g}^{2}$) and residual effects ($\sigma_{e}^{2}$).

$$\mathrm{var}\left(Y\right)={A\sigma }_{g}^{2}+{I\sigma }_{e}^{2}$$

where A is GRM with each cell representing pair‐wise genetic relatedness and I is identity matrix, assuming independence of environmental influence and no gene‐gene or gene‐environment interaction. For example, A_jk_ represents genetic relatedness between individuals j and k from m genotyped SNPs:

$$A_{\mathrm{jk}}=\frac{1}{m}\Sigma_{m}\frac{\left(x_{\mathrm{ij}}-2p_{i}\right)\left(x_{\mathrm{ik}}-2p_{i}\right)}{2p_{i}\left(1-p_{i}\right)}$$

where p_i_ is the minor allele frequency (MAF) and x_i_ is the genotype code of the SNP i (x_i_ = 0, 1, or 2).

A limitation of the GCTA approach is that it relies heavily on LD between assayed and causal variants (Speed et al., 2012; Zhu & Zhou, 2020). Therefore, it overestimates and underestimates the contribution of causal variants in high LD (strong LD between ungenotyped causal and genotyped variants) and low LD (weak LD between ungenotyped causal and genotyped variants) regions, respectively. In addition, genetic relatedness between a pair of individuals based on genotyped variants may not reflect genetic relatedness based on ungenotyped causal variants. If ungenotyped causal variants are in strong LD as compared to genotyped variants, heritability estimated using genotyped variants will be underestimated. GCTA suggests a uniform transformation of relatedness matrix [scaling the genotype matrix with 2p(1‐p)^–1^, where p is MAF]. Such scaling implies that effect sizes are inversely proportional to MAF and each causal variant contributes equally to the phenotypic variance. However, equal contribution of each causal variant to the phenotypic variance is not realistic due to uneven LD patterns across the genome. Additionally, assortative mating, epistasis, and gene‐environment interaction can bias heritability estimates by incorrectly allocating variance due to these phenomena to additive genetic effects. Likewise, population structure (admixed population) can bias the estimation of heritability. This bias can usually be avoided by identifying population structure through principal component analysis (PCA) and eliminating outliers from the data or correcting for admixed samples in the analysis by including the first few PCs as fixed effects in the LMM.

Later, several other variants of GCTA based on MAF stratified variants (GCTA‐MS), LD and MAF stratified variants (GCTA‐LDMS), were developed to overcome these limitations (see Alternate Protocol 1). These approaches facilitated not only partitioning of genetic variance into additive and non‐additive components, but also variance attributed to different chromosomes, genes and inter‐genic regions, biological pathways, and SNP functions (Yang et al., 2015; Yang, Manolio, et al., 2011). In addition, an approach was introduced to estimate SNP‐heritability in individuals with close or extended relationships (Zaitlen et al., 2013). This approach essentially uses GREML with two GRMs; the first GRM is created using all SNPs, whereas a threshold is applied on the second GRM by setting off‐diagonals < threshold to zero (see Basic Protocol 3). However, each approach has advantages and disadvantages (Table 1).

#### Linkage Disequilibrium Adjusted Kinships (LDAK)

Speed et al. (2012) developed a method (Linkage Disequilibrium Adjusted Kinships; LDAK) to overcome the bias arising from ungenotyped causal variants in regions of high or low LD. Yang and colleagues suggested a uniform scaling of the SNP‐based kinship matrix [2p(1‐p)^–1^, where p is MAF]. This transformation adjusts for the average bias caused by variable LD leading to uneven tagging of ungenotyped causal SNPs across the genome; however, it depends upon the MAF spectrum of the causal SNPs, which is generally not known. In contrast, LDAK suggests modification of the GRM according to local LD—contribution of the SNPs to the genetic similarity between a pair of individuals is weighted according to the LD with their neighboring SNPs. Estimating heritability using genetic similarity adjusted for local LD reduces the potential bias and increases the precision of the heritability estimate.

Reanalysis of the height data with LD‐adjusted GRM showed a slight change in the estimated SNP‐based heritability (${\hat{h}}_{\mathrm{SNP}}^{2}$), suggesting that the underestimation of h^2^ in low‐LD regions was balanced by overestimation of h^2^ in high‐LD regions. However, approximately a quarter increase in ${\hat{h}}_{\mathrm{SNP}}^{2}$ of hypertension and type I diabetes using LDAK as compared to GCTA suggested that causal SNPs were poorly tagged by genotyped SNPs—i.e., causal SNPs had lower MAF than genotyped SNPs, and a uniform transformation did not represent these LD patterns (Speed et al., 2012). In contrast, nearly one tenth decrease in ${\hat{h}}_{\mathrm{SNP}}^{2}$ of rheumatoid arthritis suggested that causal SNPs had higher MAF as compared to genotyped SNPs (Speed et al., 2012). Further, LDAK was used for imputed data across 19 human traits to develop a model for accurately describing the variation in ${\hat{h}}_{\mathrm{SNP}}^{2}$ with MAF, LD, and genotype certainty. The improved model led to on average 43% (S.D. 3%) higher ${\hat{h}}_{\mathrm{SNP}}^{2}$ than those obtained from GREML and 25% (S.D. 2%) higher ${\hat{h}}_{\mathrm{SNP}}^{2}$ than those from GREML‐LDMS (Speed et al., 2017).

Like GCTA, LDAK estimates are highly sensitive to MAF of causal variants, population stratification, and SNP data type [arrays, imputed or Whole Genome Sequence (WGS)]. In addition, as LD is a function of MAF, the weighting strategy in LDAK can introduce MAF bias because it gives more weight to SNPs with lower MAF. An analysis using all SNPs from WGS data showed that LDAK weighted SNPs inversely proportional to their LD, which resulted in near‐zero weights for common SNPs and very high weights for rare SNPs. This led to underestimated $h_{\mathrm{SNP}}^{2}$ for common variants and overestimated $h_{\mathrm{SNP}}^{2}$ for rare variants. The LDAK‐induced MAF bias can be substantial, especially when there are several rare variants, leading to an inflated estimate of $h_{\mathrm{SNP}}^{2}$(Yang et al., 2017). LDAK assumes that the variance explained by rare variants is very large in comparison to that explained by common variants, which predicts that the power to detect rare variants would be higher than that to detect common variants in the same order. This prediction is not consistent with empirical results in the cases of human height, schizophrenia, and type 2 diabetes (Yang et al., 2017).

### Approaches Utilizing Summary Results From Previous GWAS

We discussed the approaches to estimate $h_{\mathrm{SNP}}^{2}$ from individual‐level genome‐wide SNPs data; however, availability of such data with relevant phenotype information is often limited. Therefore, other approaches were developed that utilize summary results of GWAS (estimated SNP effects and their standard errors for hundreds of thousands of SNPs analyzed in a study) instead of per‐individual genome‐wide information.

#### LD Score (LDSC) regression

In GWAS, the deviation of observed χ^2^ test statistic for an SNP from its expected value under the null hypothesis (no association) is a function of LD between a target SNP and underlying causal variants (Yang et al., 2017). Therefore, $h_{\mathrm{SNP}}^{2}$ can be directly estimated from the summary results by regressing the observed χ^2^ test statistic against LD score of genome‐wide SNPs. This is the basic principle of the LD Score regression approach (LDSC; Bulik‐Sullivan et al., 2015; Bulik‐Sullivan et al., 2015). Under the polygenic model, average variance explained by each SNP is $h_{\mathrm{SNP}}^{2}$/m, where m and $h_{\mathrm{SNP}}^{2}$ are number of SNPs and total variance attributable to all SNPs in the summary data, respectively. Therefore, the expected χ^2^ can be represented as $E\left(\chi 2|l_{i}\right)=1\;+\;\frac{h_{\mathrm{SNP}}^{2}N}{m}l_{i}$ for SNP i, where N is number of individuals and l_i_ is the sum of LD r^2^ values between SNP i and all SNPs (including itself). This approach requires only summary data from GWAS because LD scores can be estimated in a reference population (for example, the 1000 Genomes Project). Besides LD between the target SNP and the underlying causal variants, cryptic relatedness/population stratification can also inflate the expected χ^2^ test statistics. Therefore, an extra term (a) can be included in the model for confounding biases: $E\left(\chi 2|l_{i}\right)=1+\mathrm{Na}+\;\frac{h_{\mathrm{SNP}}^{2}N}{m}l_{i}$. Regression of the observed χ^2^ test statistics against LD scores of genome‐wide SNPs [$E\left(\chi 2|l_{i}\right)=b_{0}+b_{1}l_{i}$] can not only detect an inflation due to confounding factors such as population stratification (b_0_ >1 represents confounding bias) but also estimate $h_{\mathrm{SNP}}^{2}$ (${\hat{h}}_{\mathrm{SNP}}^{2}=\frac{b_{1}m}{N}$).

Like GCTA, LDSC has also been extended to estimate genetic correlation (r_g_) between traits using summary results. Genetic correlation can be defined as genetic covariance normalized by SNP‐heritability [$r_{g}=\frac{\rho_{xy}}{\sigma_{x}\sigma_{y}}\approx \frac{h_{xy}^{2}}{\sqrt{h_{x}^{2}h_{y}^{2}}}$], where $\rho_{xy}$, $\sigma_{x}$, and $\sigma_{y}$ represent genetic covariance between trait x and y, the square root of genetic variance of x and y, respectively, which can be approximated as additive genetic covariance between x and y ($h_{xy}^{2}$) and square roots of SNP‐based narrow‐sense heritability of x ($h_{x}^{2}$) and y ($h_{y}^{2}$). Unlike pedigree‐based methods, bivariate (multivariate) GREML, an extension of GREML, estimates genetic correlations between traits (or diseases) in unrelated individuals, for example two independent studies. While LDSC also allows the traits sought for genetic correlation to be measured on different sets of samples, a major advantage of LDSC is that it requires only summary statistics. Calculations for genetic correlation are quite similar to heritability estimations via LDSC. χ^2^ statistics for a single study are replaced with the product of z‐scores from two studies of traits with non‐zero genetic correlation. An expected value of product of z‐scores from two studies, study 1 and 2, based on SNP i can be expressed as $E\left(z_{1i}z_{2i}\right)=\frac{\rho N_{S}}{\sqrt{N_{1}N_{2}}}+\frac{\sqrt{N_{1}N_{2}}\rho_{g}}{m}l_{i}$, where N_1_ and N_2_ are sample sizes from study 1 and 2, N_S_ is the number of individuals included in both studies, ρ is the phenotypic correlation among the N_S_ overlapping samples, ρ_g_ is the genetic covariance between trait 1 and 2, and l_i_ is the sum of LD r^2^ values between SNP i and all SNPs (including itself). Hence, ρ_g_ can be estimated by regressing the product of z‐scores from two studies (z_1_z_2_) on LD r^2^ values (${\hat{\rho }}_{g}=\frac{b_{1}m}{\sqrt{N_{1}N_{2}}}$). If study 1 and study 2 are the same study, then genetic covariance between a trait and itself is the estimate of heritability (${\hat{h}}_{\mathrm{SNP}}^{2}$), and χ^2^=z^2^. Once genetic covariance (ρ_g_) is estimated, genetic correlation (r_g_) can be estimated in the same way as with GCTA. As compared to GCTA, LDSC provides great flexibility to estimate r_g_ between any two GWAS data sets.

A major advantage of LDSC is that it is faster than individual‐based approaches and its computing time does not scale up with sample size. LDSC only requires summary data, which allows the reanalysis of summary data available from published meta‐analyses. The LD score regression intercept can be used to estimate population stratification. Since summary results are available usually only for common variants, LDSC is limited in estimation of the variance explained by rare variants even with imputed or WGS data, and it is more sensitive to the genetic architecture of a trait. The estimates using LDSC depend on the LD scores and, thereby, the reference population in which LD scores were calculated. If there is a mismatch between the LD scores from the reference population and the target population used for GWAS, then LD score regression can be biased. A previous study showed that ${\hat{h}}_{\mathrm{SNP}}^{2}$ measures from LDSC are consistently smaller than those from GREML in the same data set, which is likely due to errors in LD scores estimated from the reference population (LDSC recommends using LD scores from HapMap 3 SNPs in the 1000 Genomes Project; Ni et al., 2018).

LD score regression has been frequently applied to summary statistics from GWAS—to estimate the SNP‐heritability of a trait, average bias due to confounding, heritability enrichments of SNP categories, and genetic correlation between a pair of traits. Like GCTA, LDSC also assumes that all causal variants contribute equally to the phenotypic variance, and therefore provides equal weight to each SNP. Although this model is widely used in statistical genetics, it usually underestimates the average ${\hat{h}}_{\mathrm{SNP}}^{2}$ in regions of high LD (due to multiple tagging of causal variants). LDSC tends to over‐estimate confounding bias, under‐estimate SNP heritability, and produce exaggerated estimates of enrichments, due to misspecification of heritability model (Speed & Balding, 2019).

#### SumHer

Speed et al. (2019) proposed an approach (SumHer) to overcome the limitations of LDSC (Speed & Balding, 2019; Speed et al., 2020). The basic idea behind SumHer is that SNP heritability (e.g., ${\hat{h}}_{i}^{2}$ for SNP i) varies across the genome, and therefore, $E\left({\hat{h}}_{i}^{2}\right)\propto q_{i}={\left[p_{i}\left(1-p_{i}\right)\right]}^{1+\alpha }w_{i}$, where p_i_ is MAF of SNP i, w_i_ is the weight calculated for SNP i based on local LD, and α is a constant (like LDAK, SumHer chooses α=−0.25 by default)]. The main difference between LDSC and SumHer is that, unlike LDSC (which assumes all q_j_ are same; q_i_=1), SumHer allows users to choose any heritability model. i.e., the user can specify arbitrary values for q_i_. As compared to additive modeling of inflation in LDSC, SumHer models inflation of test statistics multiplicatively. A recent analysis of 24 large‐scale GWAS using the recommended model in SumHer showed that these studies were inclined to over‐correct for confounding, which reduced the discovery of genome‐wide significant loci by about a quarter (Speed & Balding, 2019). Heritability estimate enrichment analyses using LDSC concluded that heritability is highly concentrated in specific functional categories. For example, an analysis across 17 diseases showed that conserved regions contribute 35% of SNP heritability, indicating that they were 13‐fold‐enriched for casual variants. By contrast, analyses across 24 traits using SumHer finds that none of the categories have enrichment above 2‐fold (Speed & Balding, 2019).

SumHer proposes a solution to unequal contribution of ${\hat{h}}^{2}$ per SNP due to differential LD pattern, overestimation of confounding due to population stratification and exaggerated heritability enrichment due to misspecification of the heritability model. However, it also suffers from limitations in common with LDSC. It is not known yet how well the SumHer heritability model would fit while estimating variance attributable to rare variants. Like LDSC, heritability estimates from SumHer depend on LD scores from reference samples indicating a mismatch in LD scores from reference samples and GWAS samples would result in biased estimation of ${\hat{h}}^{2}$.

## STEP‐BY‐STEP GUIDE FOR SNP‐HERITABILITY ESTIMATION

Here, we provide a stepwise guide to estimate SNP‐heritability using various approaches. For illustration purposes, we use individual‐level genetic data and summary results from previous GWAS to estimate SNP‐heritability of height and BMI. Individual‐level genetic data from The Northern Finnish Birth Cohort (NFBC; 1966) consists of several metabolic trait measurements in 5402 individuals (Sabatti et al., 2009), genotyped for 364,580 SNPs using the Illumina HumanCNV370‐Quadv3_C platform. Likewise, we use summary results from meta‐analysis of height and BMI using UK Biobank and GIANT GWAS (2018) (Yengo et al., 2018).

The NFBC dataset is available through DbGaP authorized access. These data are for general research use—i.e., use of the data is limited only by the terms of the model Data Use Certification. There is no limitation in the usage of the genomic results outside the study for which they were originally consented. Summary results from the GIANT consortium are publicly available and can be accessed without restriction.

Although not an integral part of the protocol, we also provide a brief overview of widely used quality control procedure for phenotype and genotype data. Current approaches, developed for SNP‐heritability estimation can also be used for various other purposes for example, estimation of genetic correlation, confounding due to population structure and cryptic relationship, gene enrichment analysis. However, we provide protocols only for the SNP‐heritability estimation to align with the focus of the current review.

### Resources

Before starting the estimation of SNP‐heritability, it is necessary to install appropriate software; assemble the data files; input genotype (usually, plink format is preferred; if genotypes are present in variant call format (vcf) file, it can be converted to plink format for further analyses), phenotype (phenotype file should have at least three columns in the order family id, individual id, and phenotypic values), and summary results (usually contains an identifier/SNP id, effect allele, other allele, sample size, *p* value, and summary statistics); and download pre‐calculated tagging (LD score) information from reference population (e.g., 1000 Genomes database, UK Biobank database). Reference population used for LD scores should be ancestrally similar to the GWAS samples. Similarly, we should be cautious while using summary results from previous GWAS and use the large studies with rigorous quality control.

#### Hardware

Any laptop/computer with 4 cores and 8‐16 GB RAM is sufficient for most of the analyses in a reasonable time (Yang, Lee, et al., 2011).

#### Operating system

Linux‐based operating system such as Ubuntu, Fedora etc.

### Quality Control of Phenotype and Genotype Data

A detailed description of quality control for genome‐wide analysis can be found elsewhere (Truong et al., 2022; Turner et al., 2011; Weale, 2010). Here, we briefly summarize the routinely used quality control procedures. In general, quality control of phenotypes depends on the research question, trait type (discrete or continuous), and other phenotypes/covariates available in the dataset. R (R Team, 2020)/R‐Studio (R Team, 2019) (https://www.R‐project.org) is a well‐established platform for phenotype quality control. The first step is to select a key phenotype in a given dataset with multiple phenotypes, followed by summarizing the data. A density plot for continuous traits and bar plot for discrete traits can provide a rough idea about phenotype distribution and outliers. As normal distribution of variables is one of the assumptions in commonly used analyses, removing outliers (mean ±4*S.D.) is a common practice to attain normality in the dataset. However, one should be cautious while removing outliers and should choose this option only when alternative approaches such as data transformation (log or exponential) or adjusting with other covariates do not work. Phenotype data can be supplied to linear models either adjusted for the covariates or without adjusting for covariates where covariates can be supplied separately into the model.

Quality control of the genotype file is performed based on individuals and markers. PLINK (Chang et al., 2015; Purcell et al., 2007) (https://www.cog‐genomics.org/plink/1.9) and R/R‐Studio (R Team, 2019; R Team, 2020) (https://www.R‐project.org) are routinely used for genotype quality control and plotting various quality measures, respectively. In general, individuals are filtered on the basis of genotype missing rate, average heterozygosity (inbreeding), inconsistency between biological and reported sex, and Mendelian errors (if pedigree information is available). Similarly, markers are filtered on the basis of call rate, minor allele frequency (MAF), and Hardy‐Weinberg equilibrium (HWE). In addition, heritability estimation methods assume a homogeneous population; therefore it is advisable to check for population stratification and remove outliers using principal component analysis (PCA) or multi‐dimensional scaling (MDS) based on the set of markers in the genome that are independent.

We performed the quality control procedure for NFBC dataset as mentioned above. After careful examination of the density plot of height and BMI, 33 samples were removed from the analysis. Phenotypes were adjusted for sex before fitting into LMM. Similarly, genotype data were controlled for individual and marker quality. Individuals were examined and excluded on the basis of genotype missing rate >5%, average heterozygosity ± 4*S.D., and inconsistency between reported and biological sex, whereas SNPs were examined and excluded on the basis of call rate <95%, MAF <1%, and HWE with *p* < 1.0E‐6. After genotype and phenotype quality control, 5348 individuals genotyped on 324,851 autosomal SNPs with available phenotype information remained for SNP‐heritability estimation analyses.

### Protocols

Assuming that genotype and phenotype files are pre‐processed for quality control, we provide the protocols below to run SNP‐heritability analyses using different heritability estimation approaches. We believe that these protocols will make it easy for readers to estimate SNP‐heritability ($h_{\mathrm{SNP}}^{2}$) using individual‐level data and summary statistics.

## Basic Protocol 1 GREML (GCTA)

This GREML protocol can be broadly categorized into three steps—(1) create genetic relatedness matrix (GRM); (2) remove one of the cryptically related individual pairs; (3) run restricted maximum likelihood (REML). GCTA allows multi‐threading that can be enabled by using the flag ‐‐thread‐num or ‐threads.

### Software and files needed for GREML

#### Software

GCTA (Yang et al., 2010; Yang, Lee, et al., 2011; https://yanglab.westlake.edu.cn/software/gcta/#Download)

##### Data file

The Northern Finland Birth Cohort (Sabatti et al., 2009; https://www.ncbi.nlm.nih.gov/projects/gap/cgi‐bin/study.cgi?study_id=phs000276.v2.p1)

1 Create GRM using plink format files (test.bed, test.bim, and test.fam).
  Depending upon the requirements of the analysis, GRMs can be created in different ways, such as by using only autosomes, using each chromosome separately, using the X chromosome alone, or using a subset of SNPs.
  a Using autosomes only:

gcta64 ‐‐bfile test ‐‐autosome ‐‐make‐grm‐bin ‐‐out test_grm ‐‐thread‐num 4;

  b Based on each chromosome separately:

gcta64 ‐‐bfile test ‐‐chr 1 ‐‐make‐grm‐bin ‐‐out test_grm_chr1 ‐‐thread‐num 4;

gcta64 ‐‐bfile test ‐‐chr 2 ‐‐make‐grm‐bin ‐‐out test_grm_chr2 ‐‐thread‐num 4;

. . .

gcta64 ‐‐bfile test ‐‐chr 22 ‐‐make‐grm‐bin ‐‐out test_grm_chr22 ‐‐thread‐num 4;

  c Using X chromosome.

gcta64 ‐‐bfile test ‐‐make‐grm‐xchr ‐‐out test_grm_xchr ‐‐thread‐num 4;

  d Create GRM with a subset of SNPs (test_snplist.txt *—one SNP on a line*)

gcta64 ‐‐bfile test ‐‐extract test_snplist ‐‐make‐grm‐bin ‐‐out test_grm_subset ‐‐thread‐

num 4;

2 Remove one individual from each cryptically related pair using kinship coefficient cutoff (0.05):

gcta64 ‐‐grm test_grm ‐‐grm‐cutoff 0.05 ‐‐make‐grm‐bin ‐‐‐out test_grm_0.05 ‐‐thread‐num 4;

3 Run REML with kinship matrix (test_grm_0.05.grm.bin, test_grm_0.05.grm.N.bin, and test_grm_0.05.grm.id) and phenotype file (test.phen):

gcta64 ‐‐grm test_grm_0.05 ‐‐pheno test.phen ‐‐reml ‐‐out test_greml ‐‐thread‐num 4;

  The phenotype file typically has three columns—Family ID, Individual ID, and Phenotype. However, more than one phenotype can also be provided and assigned to a specific column (phenotype) for $h_{SNP}^{2}$ estimation by providing an additional option ‐‐mpheno [(column‐number) ‐ 2] in the above command.

REML can also be run in various alternative ways such as using GRMs created by a subset of SNPs, using multiple GRMs, adjusting for covariates and using discrete outcomes e.g., case‐control status in phenotype file:

- Run REML using GRM created by a subset of SNPs (test_grm_subset.grm.bin, test_grm_subset.grm.N.bin, and test_grm_subset.grm.id):

gcta64 ‐‐grm test_grm_subset ‐‐keep test_grm_0.05.grm.id ‐‐pheno test.phen ‐‐reml ‐‐out

test_greml_subset ‐‐thread‐num 4;

- Run REML using multiple GRMs (grm_chrs.txt is a text file with list of GRM names—one GRM name on a line):

gcta64 ‐‐mgrm grm_chrs.txt ‐‐pheno test.phen ‐‐reml ‐‐out

test_greml_chrs ‐‐thread‐num 4;

- Adjust for covariates (‐‐covar and ‐‐qcovar for discrete and continuous covariates, respectively):

gcta64 ‐‐reml ‐‐grm test_grm_0.05 ‐‐pheno test.phen ‐‐covar sex.txt ‐‐qcovar PCs.txt ‐‐out

test_greml_adj ‐‐thread‐num 4;

sex.txt is a list of individuals’ sexes (discrete variable) and PCs.txt is a file with first 10‐20 principal components (continuous variable). Similar to the phenotype files, covariate files also have the first two columns as family id and individual id followed by covariate columns.
- Run REML for case control data **(** test_cc.phen —phenotype file with case‐control information). Let us assume that the prevalence of the disease is 0.1 in the general population. The option ‐‐prevalence is used to specify the disease prevalence and transformation of ${\hat{h}}_{\mathrm{SNP}}^{2}$ from observed discrete (0‐1) scale to unobserved continuous liability scale.

gcta64 ‐‐reml ‐‐grm test_grm_0.05 ‐‐pheno test_cc.phen ‐‐prevalence 0.1 ‐‐out

test_greml_cc ‐‐thread‐num 4;

Usually, GCTA runs REML in a constrained manner such that 0 < ${\hat{h}}^{2}$ < 1. If there are multiple matrices each with a small contribution to ${\hat{h}}^{2}$, one or more random effects may hit the boundary. REML stops if more than half of the total components hit the boundary. To avoid such situation, ‐‐reml‐no‐constrain can be used to run REML in an unconstrained manner.

## Alternate Protocol 1 STRATIFIED GREML

As seen in the previous example, SNP‐heritability attributable to each chromosome can be estimated by simultaneously fitting GRMs based on each chromosome in to REML. Similarly, GREML can be run in various other stratified ways, for example, using GRMs created by a subset of SNPs stratified by either minor allele frequency (MAF) bins alone or both linkage disequilibrium (LD) and MAF bins. These variations of GREML were developed to adjust for the influence of MAF and local LD on the estimated SNP‐heritability, and known as the GREML‐MAF Stratified (GREML‐MS) and GREML‐LD and MAF Stratified (GEML‐LDMS) approach, respectively. Like original GREML, stratified GREML is also performed in three major steps: (1) create GRM, (2) remove one of the cryptically related individual pairs, and (3) run REML. However, GREML‐LDMS includes an additional step—calculation of LD scores (summation of r^2^ values between a SNP and all SNPs in a given genomic region) prior to creating GRMs. It is noteworthy that multiple GRMs are created and fitted in REML based on the stratification criteria in stratified GREML.

### Software and files needed for Stratified‐GREML

#### Software

GCTA (Yang et al., 2010; Yang, Lee, et al., 2011)) (https://yanglab.westlake.edu.cn/software/gcta/#Download)
R/R‐Studio (R Team, 2019; R Team, 2020; https://www.R‐project.org)

##### Data file

The Northern Finland Birth Cohort (Sabatti et al., 2009; https://www.ncbi.nlm.nih.gov/projects/gap/cgi‐bin/study.cgi?study_id=phs000276.v2.p1)

#### GREML‐MS (Based on MAF bins only)

1a Create GRMs:

gcta64 ‐‐bfile test ‐‐autosome ‐‐maf 0.01 ‐‐max‐maf 0.1 ‐‐make‐grm‐bin ‐‐out

test_maf0.1_grm ‐‐thread‐num 4;

gcta64 ‐‐bfile test ‐‐autosome ‐‐maf 0.1 ‐‐max‐maf 0.2 ‐‐make‐grm‐bin ‐‐out

test_maf0.2_grm ‐‐thread‐num 4;

. . .

gcta64 ‐‐bfile test ‐‐autosome ‐‐maf 0.4 ‐‐max‐maf 0.5 ‐‐make‐grm‐bin ‐‐out

test_maf0.5_grm ‐‐thread‐num 4;

2a Remove one of the cryptically related individual pairs: REML can be run with unrelated individuals by adding a flag ‐‐keep [list of individuals with kinship coefficient < threshold e.g. 0.05] . A list of individuals with kinship coefficient less than a set threshold can be created using the protocol provided in GCTA. Alternatively, the test_grm_0.05.grm.id file created in the GREML protocol can directly be used. It is noteworthy that stratified REML analysis is performed with multiple GRMs listed in a text file (one GRM name in a line).
3a Run REML:

gcta64 ‐‐mgrm greml_ms_grm_list.txt ‐‐pheno test.phen ‐‐reml ‐‐out test_greml_ms ‐‐thread‐num 4;

#### GREML‐LDMS (based on LD and MAF bins)

1b Calculate LD scores:
  LD scores are calculated using option ‐‐ld‐score‐region **[window size]** . GCTA uses default window size of 200 Kb with 100Kb overlapping regions between two segments:

gcta64 ‐‐bfile test ‐‐autosome ‐‐ld‐score‐region 200 ‐‐out test_ld ‐‐thread‐num 4;

  Import the output of the above command ( test_ld.score.ld ) to R and create quartiles based on either ldscore_SNP or ldscore_region . Save SNPs corresponding to each quartile as test_ld_q*.txt , where * is 1/2/3/4. Different bins are created on the basis of LD score quartiles and MAF ranges. For example, SNPs within each MAF range such as 0.01 < MAF ≤ 0.1, 0.1 < MAF ≤ 0.2,, 0.2 < MAF ≤ 0.3,, 0.3 < MAF ≤ 0.4 and 0.4 < MAF ≤ 0.5 can be binned on the basis of quartiles of regional or SNP LD scores.
2b Create GRM:

for i in $(seq 1 4); do

gcta64 ‐‐bfile test ‐‐autosome ‐‐extract test_ld_q${i}.txt ‐‐maf 0.01 ‐‐max‐maf 0.1 ‐‐make‐

grm‐bin ‐‐out test_q${i}_maf0.1_grm ‐‐thread‐num 4;

done;

for i in $(seq 1 4); do

gcta64 ‐‐bfile test ‐‐autosome ‐‐extract test_ld_q${i}.txt ‐‐maf 0.1 ‐‐max‐maf 0.2 ‐‐make‐

grm‐bin ‐‐out test_q${i}_maf0.2_grm ‐‐thread‐num 4;

done;

. . .

for i in $(seq 1 4); do

gcta64 ‐‐bfile test ‐‐autosome ‐‐extract test_ld_q${i}.txt ‐‐maf 0.4 ‐‐max‐maf 0.5 ‐‐make‐

grm‐bin ‐‐out test_q${i}_maf0.5_grm ‐‐thread‐num 4;

done;

3b Remove one of the cryptically related individual pairs: One individual from the cryptically related pairs can be removed using the command provided in the GCTA protocol. Alternatively, an already filtered list of unrelated individuals can be used as in GREML‐MS.
4b Run REML:

gcta64 ‐‐mgrm greml_ldms_grm_list.txt ‐‐pheno test.phen ‐‐reml ‐‐out test_greml_ldms ‐‐thread‐num 4;

## Basic Protocol 2 LDAK

This LDAK protocol can be divided into five steps—(1) Thinning of SNPs; (2) calculating weights of thinned SNPs based on the pair‐wise LD with all nearby SNPs in a bin (e.g., 100 kb); (3) creating kinship matrix; (4) removing one of the cryptically related individual pairs; (5) running REML. In the following protocols, we use a default setting of α = −0.25; the user may change this depending on the desired model. Like GCTA, LDAK also allows multi‐threading for most of the analyses which can be enabled by using the option ‐‐max‐threads.

### Software and files needed for LDAK

#### Software

LDAK (Speed et al., 2012, 2017) (http://dougspeed.com/downloads) for the first‐time users and (http://dougspeed.com/downloads2) for the returning users

##### Data file

The Northern Finland Birth Cohort (Sabatti et al., 2009; https://www.ncbi.nlm.nih.gov/projects/gap/cgi‐bin/study.cgi?study_id=phs000276.v2.p1)

1 Thinning of SNPs:
  Thinning of SNPs means removing one of the SNP pairs that are in strong LD with each other from the analysis. LDAK uses r^2^ = 0.98 and 100 Kb window size as default values:

ldak5.1.linux ‐‐thin ‐‐bfile test ‐‐chr AUTO ‐‐window‐prune .98 ‐‐window‐kb 100;

awk '{print $1, 1}' thin.in > weights.thin;

All thinned SNPs in the file weights.thin are assigned equal weight, i.e., 1, and are used for calculation of kinship matrix using LDAK‐Thin model.
2 Calculate weights of thinned SNPs:
  All the thinned SNPs are weighted equally for the LDAK‐Thin model, whereas variant specific weights are calculated for the LDAK model. Prior to calculation of variant specific weights, LDAK cuts the thinned SNPs into multiple sections. We save these sections and corresponding SNP weights for each chromosome in a sub‐directory ./sections/section${j}, where j represents chromosome number 1‐22.

awk 'NR==FNR{x[$1] = $0; next} $2 in x{print $1 ":" $2 ":" $4 ":" $6 ":" $5}' thin.in test.bim > extend_thin.in;

awk 'NR==FNR{x[$1] = $0; next} $2 in x{print $1 ":" $2 ":" $4 ":" $6 ":" $5}' thin.out test.bim > extend_thin.out;

for j in $(seq 1 22); do

mkdir ‐p ./sections/sections$j/;

awk ‐v var=$j '{split($1, a, ":"); if(a[1] == var) print a[2]}' extend_thin.in >

./sections/sections$j/thin.in;

done;

for j in $(seq 1 22); do

awk ‐v var=$j '{split($1, a, ":"); if(a[1] == var) print a[2]}' extend_thin.out >

./sections/sections$j/thin.out;

done

for j in $(seq 1 22); do

ldak5.1.linux ‐‐cut‐weights ./sections/sections$j ‐‐bfile test ‐‐chr $j ‐‐no‐thin DONE ‐‐

max‐threads 4;

ldak5.1.linux ‐‐calc‐weights‐all ./sections/sections$j ‐‐bfile test ‐‐chr $j ‐‐max‐threads

4;

done;

cat ./sections/sections{1..22}/weights.short > ./sections/weights.short;

  Thinned SNPs in the file weights.short have SNP‐specific weights and are used to calculate kinship matrix using the LDAK model. weights.short usually has a smaller number of SNPs than initially thinned SNPs because many of the thinned SNPs have zero weight and are not included in the calculation of kinship matrix.
3 Create kinship matrix.
  a. Calculate Kinship matrix using same weight for all thinned SNPs (LDAK‐Thin model):

ldak5.1.linux ‐‐calc‐kins‐direct test_grm_ldak_thin ‐‐bfile test ‐‐chr AUTO ‐‐weights

weights.thin ‐‐power ‐0.25 ‐‐max‐threads 4;

  b. Calculate Kinship matrix using SNP specific weights (LDAK Model):

ldak5.1.linux ‐‐calc‐kins‐direct test_grm_ldak ‐‐bfile test ‐‐chr AUTO ‐‐weights

./sections/weights.short ‐‐power ‐0.25 ‐‐max‐threads 4;

4 Remove one of the cryptically related individual pairs.
  a. LDAK‐Thin model:

ldak5.1.linux ‐‐filter test_ldak_thin_0.05 ‐‐grm test_grm_ldak_thin ‐‐max‐rel 0.05 ‐‐max‐

threads 4;

  b. LDAK model:

ldak5.1.linux ‐‐filter test_ldak_0.05 ‐‐grm test_grm_ldak ‐‐max‐rel 0.05 ‐‐max‐threads 4;

  The above commands produce two files—test_ldak_thin_0.05.keep and test_ldak_thin_0.05.lose or test_ldak_0.05.keep and test_ldak_0.05.lose—depending on the selected model. While running REML we can use .keep file by adding a flag ‐‐keep [keep‐file]. However, we use the same set of individuals (test_grm_0.05) as used in the GCTA approach to maintain uniformity across different approaches.
5 Run REML.
  a. LDAK‐Thin model:

ldak5.1.linux ‐‐reml test_ldak_thin ‐‐pheno test.phen ‐‐pheno ‐‐grm test_grm_ldak_thin ‐‐

keep test_grm_0.05.grm.id ‐‐constrain YES ‐‐max‐threads 4;

  b. LDAK model:

ldak5.1.linux ‐‐reml test_ldak ‐‐pheno test.phen ‐‐pheno ‐‐grm test_grm_ldak ‐‐keep

test_grm_0.05.grm.id ‐‐constrain YES ‐‐max‐threads 4;

## Alternate Protocol 2 STRATIFIED LADK

A stratified version of LDAK can be run using already calculated weights of thinned SNPs (see LDAK protocol). Unlike, GCTA, LDAK does not allow –min‐maf or –max‐maf option along with –calc‐kins‐direct . Therefore, markers based on MAF bins should be extracted from ‘test.bim’ files and the list should be used to extract the set of markers while creating kinship matrix ( –extract list‐of‐SNPs.txt ). Since, we are using pre‐computed weights and advise one uses already pruned set of individuals (see LDAK protocol), we provide rest two steps here – i) create kinship matrix; ii) Run REML.

### Software and files needed for Stratified LDAK

#### Software

LDAK (Speed et al., 2012, 2017) (http://dougspeed.com/downloads) for the first‐time users and (http://dougspeed.com/downloads2) for returning users

##### Data file

The Northern Finland Birth Cohort (Sabatti et al., 2009; https://www.ncbi.nlm.nih.gov/projects/gap/cgi‐bin/study.cgi?study_id=phs000276.v2.p1)

#### LDAK‐Thin‐MS model

1a Create Kinship Matrix:

ldak5.1.linux ‐‐calc‐kins‐direct test_maf0.1_ldak_thin_grm ‐‐bfile test ‐‐chr AUTO ‐‐

extract test_maf0.1.txt ‐‐weights weights.thin ‐‐power ‐0.25 ‐‐max‐threads 4;

ldak5.1.linux ‐‐calc‐kins‐direct test_maf0.2_ldak_thin_grm ‐‐bfile test ‐‐chr AUTO ‐‐

extract test_maf0.2.txt ‐‐weights weights.thin ‐‐power ‐0.25 ‐‐max‐threads 4;

. . .

ldak5.1.linux ‐‐calc‐kins‐direct test_maf0.5_ldak_thin_grm ‐‐bfile test ‐‐chr AUTO ‐‐

extract test_maf0.5.txt ‐‐weights weights.thin ‐‐power ‐0.25 ‐‐max‐threads 4;

2a Run REML:

ldak5.1.linux ‐‐reml test_ldak_thin_ms ‐‐pheno test.phen ‐‐mgrm ldak_thin_ms_grm_list.txt

‐‐keep test_grm_0.05.grm.id ‐‐max‐threads 4;

#### LDAK‐MS Model

1b Create Kinship Matrix:

ldak5.1.linux ‐‐calc‐kins‐direct test_maf0.1_ldak_weights_grm ‐‐bfile test ‐‐chr AUTO ‐‐

extract test_maf0.1.txt ‐‐weights ./sections/weights.short ‐‐power ‐0.25 ‐‐max‐threads 4;

ldak5.1.linux ‐‐calc‐kins‐direct test_maf0.2_ldak_weights_grm ‐‐bfile test ‐‐chr AUTO ‐‐

extract test_maf0.2.txt ‐‐weights ./sections/weights.short ‐‐power ‐0.25 ‐‐max‐threads 4;

. . .

ldak5.1.linux ‐‐calc‐kins‐direct test_maf0.5_ldak_weights_grm ‐‐bfile test ‐‐chr AUTO ‐‐

extract test_maf0.5.txt ‐‐weights ./sections/weights.short ‐‐power ‐0.25 ‐‐max‐threads 4;

2b Run REML:

ldak5.1.linux ‐‐reml test_ldak_ms ‐‐pheno test.phen ‐‐mgrm ldak_ms_grm_list.txt ‐‐keep

test_grm_0.05.grm.id ‐‐max‐threads 4;

## Basic Protocol 3 THRESHOLD GREML

The threshold GRM approach uses two GRMs corresponding to one genetic component: a first GRM is the same as that created in GREML (without threshold) and a second GRM is created with a threshold by setting the off‐diagonals that are <0.05 to 0. Here, we do not need to remove samples based on the GRM threshold. SNP‐heritability attributable to the first kinship matrix is same as the SNP‐heritability estimated by GREML. Overall, the estimate represents pedigree‐based heritability, and h^2^ attributable to second GRM ($h_{\mathrm{Ped}}^{2}-h_{\mathrm{SNP}}^{2}$) represents h^2^ attributable to shared environment. Frist, a GRM is created using commands in the GREML protocol (except, removing one of the cryptically related individuals), and then the following steps can be used to estimate SNP and pedigree‐based heritability.

### Software and files needed for Threshold GREML

#### Software

GCTA (Yang et al., 2010; Yang, Lee, et al., 2011; https://yanglab.westlake.edu.cn/software/gcta/#Download)

##### Data file

The Northern Finland Birth Cohort (Sabatti et al., 2009; https://www.ncbi.nlm.nih.gov/projects/gap/cgi‐bin/study.cgi?study_id=phs000276.v2.p1)

1 Create GRM with threshold:

gcta64 ‐‐grm test_grm ‐‐make‐bK 0.05 ‐‐out test_grm_bK ‐‐thread‐num 4;

2 Run Threshold GREML:

gcta64 ‐‐mgrm threshold_grm_list.txt ‐‐reml ‐‐pheno test.phen ‐‐out test_Threshold ‐‐

thread‐num 4;

## Basic Protocol 4 LD SCORE (LDSC) REGRESSION

LDSC allows $h_{\mathrm{SNP}}^{2}$, the SNP heritability, to be directly estimated from the summary results by regressing the observed χ^2^ test statistic against LD score of genome‐wide SNPs. Estimation of $h_{\mathrm{SNP}}^{2}$ using LDSC can be broken down into four simple steps: i) installing the program, ii) obtaining the summary results from the study in question, iii) formatting summary results for use in LDSC, and iv) running the program to estimate common SNP heritability.

### Software and files needed for LDSC Regression

#### Software

LDSC (Bulik‐Sullivan et al., 2015; Bulik‐Sullivan et al., 2015; https://github.com/bulik/ldsc)

##### Data files

Summary Results for height and BMI (Yengo et al., 2018; https://portals.broadinstitute.org/collaboration/giant/index.php/GIANT_consortium_data_files)
LD Scores calculated in 1000 Genomes reference data (Bulik‐Sullivan et al., 2015; https://alkesgroup.broadinstitute.org/LDSCORE)

1 Installation and activation.
  LDSC can be installed from the resource provided earlier using following command:

git clone https://github.com/bulik/ldsc.git;

  LDSC is a python package and an Anaconda environment (environment.yml) present in the original package must be created before using LDSC. It installs a list python dependency for LDSC:

conda env create ‐‐file environment.yml;

  Before running LDSC an Anaconda environment is installed as above and must be activated as below:

source activate ldsc

2 Download summary results.
  The next step is to download the summary results that can be downloaded from the resource provided above directly or using command line via *wget*.
3 Convert to LDSC recognized format.
  LDSC accepts a specific format of summary statistics with six columns—a unique identifier (rs id), allele 1 (effect allele), allele 2 (other allele), sample size, *p*‐value and a signed summary‐statistics (effect, odds ratio, log odds ratio, Z score). Sometimes sample size is not provided in the summary results. In that case, a uniform sample size can be provided by using a flag ‐‐N [sample size]. In the case of unsigned effects, LDSC assumes allele 1 to be a risk increasing/positively associated allele and processes summary result accordingly. Although summary results can be formatted manually, LDSC recommends using the python script munge_sumstats.py provided in the original package because it checks for several things besides converting summary result to LDSC format. In addition, it is recommended to use SNPs from summary results that are common in the HapMap3 dataset, particularly if the summary result is obtained from imputed data.
  HapMap SNPs (w_hm3.snplist.bz2) can be downloaded from https://data.broadinstitute.org/alkesgroup/LDSCORE/ either directly or using command line via wget.

Munge_sumstats.py ‐‐sumstats [summary‐result] ‐‐out [sumstats‐ldsc] ‐‐merge‐alleles

w_hm3.snplist.txt;

4 Estimate heritability.
  To estimate heritability attributable to common variants present in summary result, χ^2^ values from the output of above command (sumstats‐ldsc.gz) is regressed on the ld scores (sum of r^2^ values for a SNP with surrounding SNPs in a predefined window) calculated in a reference population such as the 1000 Genomes Project or UK Biobank. LD scores can be downloaded from the link provided in the resource. Assuming the GWAS included European population, LD scores should be used from European population, for example eur_w_ld_chr. In addition to LD scores, LDSC requires a regression weight file that includes r^2^ values for the SNPs used in the regression, i.e., GWAS SNPs. Generally, LDSC is not very sensitive to regression weights. Therefore, it is currently recommended to use the same LD scores for both flags. For partitioned h^2^ estimation, one may choose a subset of GWAS SNPs to calculate LD scores using 1000 Genomes data separately, and use them as regression weight.

ldsc.py ‐‐h2 [sum‐stats‐file.gz] ‐‐ref‐ld‐chr eur_w_ld_chr/ ‐‐w‐ld‐chr eur_w_ld_chr/‐‐out

out_h2;

  If the original GWAS already controlled for population stratification and cryptic relatedness, the intercept can be constrained by adding a flag ‐‐intercept‐h2 [threshold] or ‐‐no‐intercept which constrains the intercept to 1.

## Basic Protocol 5 SumHer

SumHer is integrated into LDAK software; therefore, no extra software needs to be installed. Unlike LDSC, one must modify summary results to SumHer‐compatible format manually. A compatible summary stats file has 5 or 6 columns (column names are case sensitive) with core columns: ‘Predictor’, ‘A1’, ‘A2’, ‘n’; then, there are three options to choose additional 1‐2 columns. The last column could be ‘Z’, or last two columns could be ‘Direction’, ‘Stat’ or ‘Direction’, ‘P’. Predictor should be in ‘chr:position’ format.

### Software and files needed for LDSC Regression

#### Software

LDAK (Speed et al., 2012, 2017; http://dougspeed.com/downloads) for first‐time users and (http://dougspeed.com/downloads2) for returning users

#### Data files

Summary Results for height and BMI (Yengo et al., 2018; https://portals.broadinstitute.org/collaboration/giant/index.php/GIANT_consortium_data_files)
LD Scores calculated in 2000 Great Britain samples from UK Biobank dataset; http://dougspeed.com/pre‐computed‐tagging‐files)
*There are several tagging files available. Based on the recommendation of SumHer authors, we used BLD‐LDAK tagging file (GBR population, HapMap SNPs) in our analysis*.

1 Convert summary result to SumHer compatible format.
  Let us assume height summary results were downloaded from GIANT consortium and unzipped to height_raw.txt using gunzip ‐c [summary‐result.gz] > height_raw.txt. This file can be formatted to get height summary results with specific columns needed for SumHer.

awk 'BEGIN{print "Predictor A1 A2 Direction P n"}

 (NR > 1 && ($2 == "A" || $2 == "C" || $2 == "G" || $2 == "T")

&& ($3 == "A" || $3 == "C" || $3 == "G" || $3 == "T")){print $1, $2, $3, $5, $7, $8}'

height_raw.txt > height.txt;

  Then, download the list of HapMap3 SNPs with chromosome and position information (https://www.dropbox.com/s/xabjdu6squ6u56r/hapmap3.snps) and format the first column of height.txt:

awk '(NR == FNR){a[$1] = $2; b[$1] = $3$4; next} (FNR ==1){print $0}($1 in a && ($2$3 ==

b[$1] || $3$2 == b[$1])){$1 = a[$1]; print $0}' hapmap3.snps height.txt > height_hm3.txt;

2 Estimate heritability.
  SNP tagging information must be downloaded prior to estimating heritability. LDAK has SNP tagging files pre‐calculated using LDAK‐Thin, BLD‐LDAK, and BLD‐LDAK‐Light+Alpha models in different populations. These files can be downloaded from the link provided in the resource, depending on the population used in the original GWAS. It is noteworthy that alpha values should be downloaded from (https://www.dropbox.com/s/o7xphugm4mln9xa/pow.txt) for using BLD‐LDAK‐Light+Alpha model. This model is useful for gene enrichment analysis. Once SNP tagging information is downloaded, SNP‐heritability can be estimated using the flag ‐‐sum‐hers.

ldak5.1.linux ‐‐sum‐hers height ‐‐summary height_hm3.txt ‐‐tagfile

bld.ldak.hapmap.gbr.tagging ‐‐check‐sums NO;

‐‐Check‐sums is a mandatory flag that tells the pipeline not to match the number of SNPs in summary result to those in the reference tagging file because, generally, all tag SNPs are not present in GWAS summary result.

## ESTIMATION OF SNP‐HERITABILITY USING INDIVIDUAL‐LEVEL DATASET AND SUMMARY RESULTS

**Figure 2:**
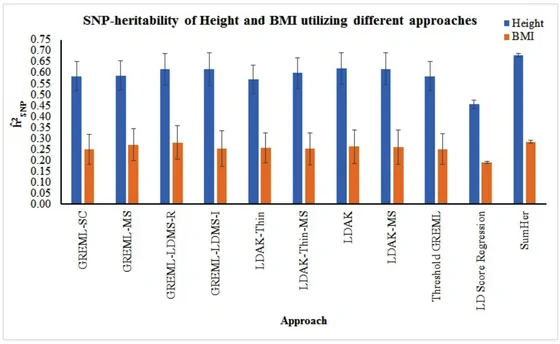
Estimation of SNP‐heritability of height and BMI using various approaches utilizing individual‐level genetic data and summary results from previous GWAS. Threshold GREML shows variance attributable to the first GRM. Stratified GREML and LDAK approaches show sum of variances attributable to all genetic components.

**Figure 3:**
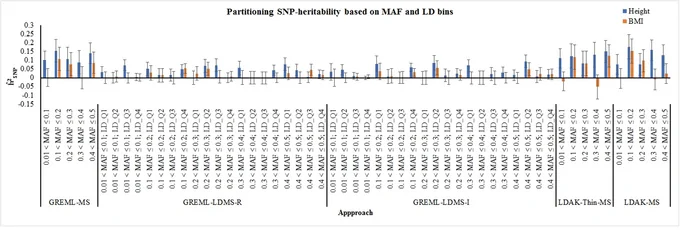
Partitioning the SNP‐heritability using MAF and LD bins. For GREML‐MS, LDAK‐Thin‐MS, and LDAK‐MS, MAF bins were created as 0.01 < MAF ≤ 0.1, 0.1 < MAF ≤ 0.2, 0.2 < MAF ≤ 0.3, 0.3 < MAF ≤ 0.4, and 0.4 < MAF ≤ 0.5. For GREML‐LDMS, each MAF bin was further divided into quartiles of average regional LD score or SNP LD score.

We compared eleven approaches for the estimation of SNP‐heritability of height and BMI utilizing individual‐level dataset (NFBC) and summary results from the GIANT consortium (Table 1). Using GREML, LDAK, and Threshold GREML approaches, we observed that genome‐wide variations explained 56.9%‐61.8% and 25%‐28.1% variance in height and BMI respectively, in NFBC (Fig. 2, Table 2). We also used stratified analysis such as stratified‐GREML and stratified‐LDAK to estimate SNP‐heritability attributable to different MAF and LD bins in NFBC. The sum of the heritability attributable to different bins was consistent with the results using single GRM (Fig. 2; Table 2). Comparison of the results from stratified‐GREML (GREML‐LDMS‐R and GREML‐LDMS‐I) and stratified‐LDAK (LDAK‐Thin‐MS, LDAK‐MS) showed that the variance attributable to different bins based on MAF and LD scores were similar in both stratified‐GREML and stratified‐LDAK approaches (Fig. 3; Table 3). Likewise, variances attributable to different MAF bins in GREML‐MS were similar to those in GREML‐LDMS‐R and GREML‐LDMS‐I (Fig. 3; Table 3). As reported previously (Evans, Tahmasbi, Vrieze, et al., 2018; Speed & Balding, 2019; Yang et al., 2017), LDSC underestimated the SNP‐heritability (Height: $h_{\mathrm{SNP}}^{2}=45.5\%,\;S.E.=1.9\%$; BMI: $h_{\mathrm{SNP}}^{2}=19.1\%,\;S.E.=0.05\%$) as compared to approaches utilizing individual‐level data (Fig. 2; Table 2). Likewise, SumHer slightly overestimated (Height: $h_{\mathrm{SNP}}^{2}=67.9\%,\;S.E.=0.08\%$; BMI: $h_{\mathrm{SNP}}^{2}=28.4\%,\;S.E.=0.08\%$) the variance attributable to the SNPs reported in GWAS summary results (Fig. 2; Table 2). The behavior of SumHer as compared to LDSC is examined in detail elsewhere (Speed & Balding, 2019).

**Table 2:** Comparison of SNP‐Heritability (${\hat{h}}_{\mathrm{SNP}}^{2}$) of Height and BMI Utilizing Widely Used Approaches^*a*^

	Height (N = 3997)			BMI (N = 3985)		
Approach	${\hat{h}}_{\mathrm{SNP}}^{2}$	S.E.	*p*‐value	${\hat{h}}_{\mathrm{SNP}}^{2}$	S.E.	*p*‐value
GREML‐SC	0.5835	0.0658	<1.11E‐16	0.2494	0.0694	1.65E‐04
GREML‐MS	0.5867	0.0671	<1.11E‐16	0.2713	0.0719	8.06E‐05
GREML‐LDMS‐R	0.6171	0.0719	<1.11E‐16	0.2811	0.0774	1.40E‐04
GREML‐LDMS‐I	0.6152	0.0743	<1.11E‐16	0.2528	0.0811	9.13E‐04
LDAK‐Thin	0.5688	0.0647	<1.11E‐16	0.2571	0.0683	8.37E‐05
LDAK‐Thin‐MS	0.5976	0.0684	<1.11E‐16	0.2527	0.0729	2.64E‐04
LDAK	0.6183	0.0710	<1.11E‐16	0.2625	0.0761	2.81E‐04
LDAK‐MS	0.6173	0.0725	<1.11E‐16	0.2599	0.0781	4.38E‐04
Threshold GRMs	0.5836	0.0656	<1.11E‐16	0.2509	0.0695	1.53E‐04
LD Score Regression	0.4552	0.0193	<1.11E‐16	0.1908	0.0053	<1.11E‐16
SumHer	0.6785	0.0077	<1.11E‐16	0.2844	0.0078	<1.11E‐16

^*a*^ N represents the number of samples used for the analyses; GREML‐SC, GREML‐MS, GREML‐LDMS‐R, GREML‐LDMS‐I, LDAK‐Thin‐MS and LDAK‐MS represent single component GREML, MAF stratified GREML, regional LD scores and MAF stratified GREML, Individual SNP LD score and MAF stratified GREML, MAF stratified LDAK‐Thin model and MAF stratified LDAK model respectively. *p*‐values were calculated using one sided z test.

**Table 3:** Comparison of SNP‐Heritability (${\hat{h}}_{\mathrm{SNP}}^{2}$) of Height and BMI Attributable to Various MAF and LD Bins Utilizing Stratified‐GREML and Stratified‐LDAK Analyses^*a*^

		Height			BMI		
Approach	Bins	${\hat{h}}_{\mathrm{SNP}}^{2}$	S.E.	*p*‐value	${\hat{h}}_{\mathrm{SNP}}^{2}$	S.E.	*p*‐value
GREML‐MS	0.01 < MAF ≤ 0.1	0.0999	0.0531	2.99E‐02	0.0021	0.0513	4.84E‐01
	0.1 < MAF ≤ 0.2	0.1531	0.0656	9.81E‐03	0.1076	0.0663	5.24E‐02
	0.2 < MAF ≤ 0.3	0.1075	0.0655	5.03E‐02	0.0766	0.0664	1.25E‐01
	0.3 < MAF ≤ 0.4	0.0878	0.0665	9.33E‐02	0.0000	0.0646	5.00E‐01
	0.4 < MAF ≤ 0.5	0.1383	0.0594	9.94E‐03	0.0851	0.0601	7.85E‐02
GREML‐LDMS‐R	0.01 < MAF ≤ 0.1; LD_Q1	0.0324	0.0312	1.50E‐01	0.0000	0.0322	5.00E‐01
	0.01 < MAF ≤ 0.1; LD_Q2	0.0000	0.0305	5.00E‐01	0.0079	0.0312	4.00E‐01
	0.01 < MAF ≤ 0.1; LD_Q3	0.0713	0.0299	8.45E‐03	0.0000	0.0291	5.00E‐01
	0.01 < MAF ≤ 0.1; LD_Q4	0.0053	0.0211	4.01E‐01	0.0014	0.0214	4.74E‐01
	0.1 < MAF ≤ 0.2; LD_Q1	0.0502	0.0395	1.02E‐01	0.0279	0.0410	2.48E‐01
	0.1 < MAF ≤ 0.2; LD_Q2	0.0143	0.0380	3.54E‐01	0.0143	0.0378	3.53E‐01
	0.1 < MAF ≤ 0.2; LD_Q3	0.0166	0.0344	3.15E‐01	0.0000	0.0354	5.00E‐01
	0.1 < MAF ≤ 0.2; LD_Q4	0.0473	0.0272	4.11E‐02	0.0530	0.0283	3.07E‐02
	0.2 < MAF ≤ 0.3; LD_Q1	0.0000	0.0379	5.00E‐01	0.0250	0.0398	2.65E‐01
	0.2 < MAF ≤ 0.3; LD_Q2	0.0668	0.0373	3.67E‐02	0.0507	0.0380	9.10E‐02
	0.2 < MAF ≤ 0.3; LD_Q3	0.0710	0.0362	2.50E‐02	0.0050	0.0354	4.44E‐01
	0.2 < MAF ≤ 0.3; LD_Q4	0.0000	0.0276	5.00E‐01	0.0081	0.0282	3.87E‐01
	0.3 < MAF ≤ 0.4; LD_Q1	0.0556	0.0383	7.37E‐02	0.0010	0.0392	4.89E‐01
	0.3 < MAF ≤ 0.4; LD_Q2	0.0001	0.0360	4.99E‐01	0.0000	0.0361	5.00E‐01
	0.3 < MAF ≤ 0.4; LD_Q3	0.0000	0.0345	5.00E‐01	0.0000	0.0345	5.00E‐01
	0.3 < MAF ≤ 0.4; LD_Q4	0.0425	0.0290	7.12E‐02	0.0000	0.0276	5.00E‐01
	0.4 < MAF ≤ 0.5; LD_Q1	0.0766	0.0358	1.62E‐02	0.0254	0.0360	2.40E‐01
	0.4 < MAF ≤ 0.5; LD_Q2	0.0436	0.0336	9.72E‐02	0.0000	0.0334	5.00E‐01
	0.4 < MAF ≤ 0.5; LD_Q3	0.0035	0.0307	4.54E‐01	0.0457	0.0319	7.56E‐02
	0.4 < MAF ≤ 0.5; LD_Q4	0.0201	0.0246	2.06E‐01	0.0157	0.0257	2.72E‐01
GREML‐LDMS‐I	0.01 < MAF ≤ 0.1; LD_Q1	0.0340	0.0462	2.31E‐01	0.0000	0.0486	5.00E‐01
	0.01 < MAF ≤ 0.1; LD_Q2	0.0462	0.0284	5.18E‐02	0.0000	0.0283	5.00E‐01
	0.01 < MAF ≤ 0.1; LD_Q3	0.0107	0.0190	2.87E‐01	0.0064	0.0192	3.70E‐01
	0.01 < MAF ≤ 0.1; LD_Q4	0.0000	0.0099	5.00E‐01	0.0050	0.0109	3.22E‐01
	0.1 < MAF ≤ 0.2; LD_Q1	0.0786	0.0452	4.11E‐02	0.0364	0.0460	2.14E‐01
	0.1 < MAF ≤ 0.2; LD_Q2	0.0071	0.0404	4.30E‐01	0.0108	0.0410	3.96E‐01
	0.1 < MAF ≤ 0.2; LD_Q3	0.0000	0.0334	5.00E‐01	0.0019	0.0336	4.77E‐01
	0.1 < MAF ≤ 0.2; LD_Q4	0.0599	0.0230	4.58E‐03	0.0309	0.0227	8.70E‐02
	0.2 < MAF ≤ 0.3; LD_Q1	0.0000	0.0374	5.00E‐01	0.0004	0.0381	4.96E‐01
	0.2 < MAF ≤ 0.3; LD_Q2	0.0854	0.0405	1.76E‐02	0.0562	0.0404	8.21E‐02
	0.2 < MAF ≤ 0.3; LD_Q3	0.0137	0.0358	3.51E‐01	0.0002	0.0380	4.98E‐01
	0.2 < MAF ≤ 0.3; LD_Q4	0.0249	0.0282	1.89E‐01	0.0156	0.0283	2.91E‐01
	0.3 < MAF ≤ 0.4; LD_Q1	0.0700	0.0334	1.82E‐02	0.0000	0.0347	5.00E‐01
	0.3 < MAF ≤ 0.4; LD_Q2	0.0000	0.0378	5.00E‐01	0.0000	0.0391	5.00E‐01
	0.3 < MAF ≤ 0.4; LD_Q3	0.0219	0.0372	2.77E‐01	0.0000	0.0379	5.00E‐01
	0.3 < MAF ≤ 0.4; LD_Q4	0.0295	0.0309	1.70E‐01	0.0000	0.0300	5.00E‐01
	0.4 < MAF ≤ 0.5; LD_Q1	0.0141	0.0314	3.27E‐01	0.0000	0.0316	5.00E‐01
	0.4 < MAF ≤ 0.5; LD_Q2	0.0938	0.0362	4.79E‐03	0.0483	0.0372	9.68E‐02
	0.4 < MAF ≤ 0.5; LD_Q3	0.0060	0.0346	4.31E‐01	0.0211	0.0358	2.78E‐01
	0.4 < MAF ≤ 0.5; LD_Q4	0.0195	0.0278	2.41E‐01	0.0196	0.0292	2.51E‐01
LDAK‐Thin‐MS	0.01 < MAF ≤ 0.1	0.1112	0.0554	2.24E‐02	−0.0201	0.0539	3.54E‐01
	0.1 < MAF ≤ 0.2	0.1243	0.0695	3.68E‐02	0.1175	0.0704	4.76E‐02
	0.2 < MAF ≤ 0.3	0.0820	0.0699	1.20E‐01	0.0822	0.0696	1.19E‐01
	0.3 < MAF ≤ 0.4	0.1306	0.0703	3.17E‐02	−0.0518	0.0692	2.27E‐01
	0.4 < MAF ≤ 0.5	0.1495	0.0630	8.82E‐03	0.1250	0.0639	2.53E‐02
LDAK‐MS	0.01 < MAF ≤ 0.1	0.0767	0.0569	8.88E‐02	−0.0034	0.0569	4.76E‐01
	0.1 < MAF ≤ 0.2	0.1769	0.0678	4.52E‐03	0.1525	0.0691	1.37E‐02
	0.2 < MAF ≤ 0.3	0.0763	0.0623	1.10E‐01	0.0970	0.0626	6.05E‐02
	0.3 < MAF ≤ 0.4	0.1576	0.0585	3.52E‐03	−0.0099	0.0591	4.33E‐01
	0.4 < MAF ≤ 0.5	0.1298	0.0570	1.14E‐02	0.0237	0.0567	3.38E‐01

^*a*^ MS, LDMS‐R, LDMS‐I represent MAF stratified, Regional LD scores and MAF stratified, Individual SNP LD score and MAF stratified, respectively; LD_Q1‐4 represent quartiles one to four based on regional or SNP LD scores. *p*‐values were calculated using one sided z test.

## CONCLUSION AND FUTURE DIRECTION

Heritability has been widely used to improve the quality of crops and farm animals, to understand the genetic basis of complex human traits and diseases, and to estimate the response of evolutionary forces such as selection in a population. However, the utility of heritability has been limited to a certain extent, mainly due to lack of appropriate data types and heritability models. Over the past decade, several approaches fitting a variety of analytical models have been developed to estimate SNP‐heritability in unrelated individuals. In the current review, we provide an overview of these approaches along with step‐by‐step protocol to run widely used approaches for SNP‐heritability estimation.

Despite advances in heritability models and availability of genome‐wide SNP information in large datasets, estimates of $h_{\mathrm{SNP}}^{2}$ are one third to two thirds of the heritability estimated through family‐based approaches. With the availability of whole genome sequence (WGS) information in large data sets, population‐based approaches can be used to estimate the variance attributable to all variants (including rare variants), which should bring estimates from the two approaches closer. Such datasets also demand development of integrative models that can fit other types of genetic variations such as structural and copy number variants into the LMM. Inclusion of rare variants and other types of variants should not only resolve the problem of incomplete tagging of causal variants, but also fill the gap of missing heritability. However, selection of appropriate heritability models for different data types and their precision still present an ongoing debate (Speed et al., 2020; Zhu & Zhou, 2020). Therefore, a consensus needs to be reached regarding heritability models for different data types. Previously, some efforts were made to estimate SNP‐heritability utilizing identity‐by‐descent information inferred from genome‐wide data(Browning & Browning, 2013; Evans, Tahmasbi, Jones, et al., 2018). In near future, we can expect new and better approaches that can estimate unbiased $h_{\mathrm{SNP}}^{2}$ from identity‐by‐descent information inferred from WGS data. Likewise, methods using pedigree data to estimate narrow‐sense heritability should account for shared environmental effects and assortative mating. Moreover, new methods to estimate heritability free of assumptions about the relationship between effect size and minor allele frequency are also required in the near future.

## AUTHOR CONTRIBUTIONS

**Amit K. Srivastava**: Conceptualization, data curation, formal analysis, investigation, methodology, resources, software, validation, visualization, writing original draft, writing review and editing; **Scott M. Williams**: writing review and editing; **Ge Zhang**: conceptualization, data curation, funding acquisition, investigation, project administration, resources, supervision, writing review and editing.

## CONFLICT OF INTEREST

Authors declare no conflict of interest.

## Data Availability

Individual‐level genetic data that support the protocol (The Northern Finland Birth Cohort) are available in dbGaP for general research use (https://www.ncbi.nlm.nih.gov/projects/gap/cgi‐bin/study.cgi?study_id=phs000276.v2.p1). Likewise, summary results are openly available in GIANT consortium datafiles (https://portals.broadinstitute.org/collaboration/giant/index.php/GIANT_consortium_data_files).
